# Transcranial magnetic stimulation intervention in Alzheimer’s disease: a research proposal for a randomized controlled trial

**DOI:** 10.1186/s13104-018-3757-z

**Published:** 2018-09-05

**Authors:** Elena M. Marron, Raquel Viejo-Sobera, María Quintana, Diego Redolar-Ripoll, Daniel Rodríguez, Maite Garolera

**Affiliations:** 1 Cognitive NeuroLab, Faculty of Health Sciences, Universitat Oberta de Catalunya (UOC), Rambla del Poblenou, 156, 08018 Barcelona, Spain; 2 Brain, Cognition and Behavior: Clinical Research, Consorci Sanitari de Terrassa, Carretera Torrebonica s/n, 08227 Terrassa, Spain; 3 Sant Llàtzer Day Hospital for Cognitive Impairment, Consorci Sanitari de Terrassa, Plaça del Doctor Robert, 6, 08221 Terrassa, Spain; 4 Neuropsychology Unit, Brain, Cognition and Behavior: Clinical Research, Consorci Sanitari de Terrassa, Carretera Torrebonica s/n, 08227 Terrassa, Spain

**Keywords:** Alzheimer’s disease, Functional connectivity, Dorsolateral prefrontal cortex, Non-invasive brain stimulation, Parietal cortex, Theta burst stimulation, Transcranial magnetic stimulation, TMS

## Abstract

**Objective:**

Alzheimer’s disease is a major health problem in our society. To date, pharmacological treatments have obtained poor results and there is a growing interest in finding non-pharmacological interventions for this disease. Transcranial magnetic stimulation (TMS) is a non-invasive technique that is able to induce changes in brain activity and long-term modifications in impaired neural networks, becoming a promising clinical intervention. Our goal is to study the benefit of individualized TMS targeting based on the patient’s functional connectivity (personalized targeting), and short duration TMS protocol, instead of current non-individualized and longer session approaches. A double blind randomized controlled trial will be conducted to assess the effects of TMS treatment immediately, 1 month, 3 months and 6 months after the end of the intervention. Fifty-four patients with a diagnosis of Alzheimer’s disease will be randomly allocated into experimental (active TMS), sham control, or conventional intervention control group. We will quantify changes in cognitive, functional, and emotional deficits in Alzheimer patients, as well as the functional connectivity changes induced by the TMS treatment.

**Results:**

We expect to demonstrate that personalized TMS intervention has a measurable positive impact in cognition, emotion, daily living activities and brain connectivity, thus representing a potential treatment for Alzheimer’s disease.

*Trial registration* The trial has been prospectively registered at ClinicalTrials.gov, identifier NCT03121066. Date of registration: 04/19/2017

## Introduction

Alzheimer’s disease (AD) is the most common form of dementia worldwide (50–70%) [1], estimating that dementia will affect 65.7 million people by the year 2030 [2]. Despite advances in the pharmacological treatment of AD, no therapies currently exist that can modify the course of the disease [3]; transcranial magnetic stimulation (TMS) applied in combination with cognitive stimulation (CS) seems a promising approach [4, 5]. TMS is able to induce changes in cortical excitability, increasing brain plasticity and facilitating the recovery and/or reorganisation of affected neural networks in pathologies causing cognitive impairment [6–10].

The most encouraging results for the use of TMS have been obtained after applying high frequency stimulation (at 10–20 Hz) to increase patient’s cortical excitability over the left dorsolateral prefrontal cortex (DLPFC) [11, 12] or bilaterally [13–15]. Improvements have been found in general cognitive performance [13, 15], functional and depression scales [13], episodic memory and processing speed [12], and language skills [11, 14]. Newly developed protocols apply stimulation over several brain regions bilaterally, concurrently with CS, during 6 months, finding medium to large effect size improvements (0.4–0.7) in neuropsychological, clinical and functional assessments up to 4.5 months [16, 17]. Similar, but sorter interventions (6 weeks) have been employed also with encouraging results [18–21].

Despite the aforementioned promising results, to date there has been no randomized controlled trial with AD patients using the intermittent theta burst stimulation (iTBS) protocol. iTBS protocol allows an increase in cortical excitability in a much shorter time than conventional repetitive TMS (3 vs. 30 min) and has been effective, for example, in improving language deficits in Parkinson’s and post-stroke aphasia patients [22, 23].

To determine the target stimulation area, all the previous studies have looked at structural aspects, but none of them used brain functional information. Location based on the functional involvement of cortical areas in relevant cortico-subcortical networks allows a much more specific and individualized treatment approach, which might be the best option in this disease [9, 24, 25]. Finally, the underlying mechanisms explaining the observed improvements (e.g. functional and/or structural brain changes) and the possible influence of genetic factors (e.g. the presence of specific ApoE alleles) have not been explored [26, 27].

Therefore, the main goal of this clinical trial is to study the benefits of individually targeted short TMS protocol combined with CS in AD. We will assess the efficacy of iTBS protocol in the improvement of cognitive, functional and emotional deficits, as well as functional brain connectivity, and explore genetic modulatory factors. We hypothesize that a 2 weeks intervention (10 sessions every working day), stimulating the DLPFC and parietal cortex (PC) of both hemispheres, combined with CS, will be more effective than CS conducted alone.

## Main text

We will conduct a randomized, double-blind, parallel clinical trial. The participants will be randomly allocated (1:1:1) to one of the three groups: (1) *experimental group*: TMS + CS; (2) *sham control group*: sham TMS + CS; (3) *non*-*TMS control group*: CS alone. The reporting of the trial outcomes will comply with the CONSORT guidelines (http://www.consort-statement.org/) for non-pharmacologic treatment [28], and it is registered in ClinicalTrials.gov (https://clinicaltrials.gov/; identifier NCT03121066).

All the necessary means for conducting the trial will be provided both by the Cognitive NeuroLab research group (Universitat Oberta de Catalunya) and by Consorci Sanitari de Terrassa.

### Sample

The sample will consist of 54 volunteer patients (18 per group), aged 60–75 years old, with a diagnosis of AD according to the NIA-AA. To ensure the maximum homogeneity of the sample in terms of severity of the symptoms and current health condition that may interfere with the diagnostic, we will apply strict inclusion and exclusion criteria (see Table 1).

**Table 1:** Inclusion and exclusion criteria

Inclusion criteria	Exclusion criteria
Mini Mental State Examination (MMSE) [29] score between 20 and 26	Lack of knowledge of Spanish or Catalan
Global Deterioration Scale (GDS) [30] score of 3 or 4	Less than 4 years of schooling
Functional independence for basic daily life activities (part B of the Blessed Scale) [31] score equal to 0	Intellectual deficiency (Premorbid IQ, vocabulary, less than 85)
Rosen Ischemia Scale less or equal to 4 [32]	No controlled medical conditions or severe mental disorders that may affect the central nervous system, including signs of increased intracranial pressure or intracranial lesions
Able to read and write	Not controlled medical conditions that may cause emergencies or convulsions (e.g.: vascular risk, cardiac malformations or arrhythmias, asthma, etc.)
Stable medical and pharmacological condition during the 3 months immediately before the start of the study	Medical history of convulsions, previous diagnosis of epilepsy, previous registry of abnormal electroencephalogram (EEG) or family history of epilepsy
Computerized tomography scan and magnetic resonance imaging (MRI) in the 12 months prior to the selection, compatible with the diagnosis of probable AD in the subjects diagnosed	Severe hearing problems or ringing in the ears (tinnitus)
Absence of clinically significant anomalies in the medical history or clinical laboratory results during the selection	Severe loss of visual acuity
Screening analyses within normal range to detect and exclude other causes of dementia in the 12 months previous to selection. Laboratory values considered are as follows: complete blood count, thyroid hormones (TSH), T4, folic acid, vitamin B12, albumin, transaminase alanine (ALT), aminotransferase aspartate (AST), gamma-glutamic transferase (GGT), sodium, potassium, urea, creatinine, and glucose while fasting	Moderate or severe depression defined as a score > 11 in the Geriatric Depression Scale (GDS) [33]
Being treated by Acetylcholinesterase Inhibitors	Presence of tremors or lack of motor control of the dominant upper limb
Willingness to undergo MRI scan	Being under pharmacological treatment with medications indicated in the security TMS guidelines [34]
Signed consent form, previously approved by the Institutional Review Board of the Consorci Sanitari de Terrassa	Drug or alcohol consumption or history of abuse in the 24 months prior to the study
	Implants of metal pieces in the head (excluding dental implants)
	Any of the following medical devices: pacemaker, implanted medication pumps, vagal nerve stimulators, deep cerebral stimulators, transcutaneous electrical stimulation units, ventriculo-peritoneal derivations, titanium plates, cochlear implants, aneurysm clips, etc.

Participants will be selected from patients attending the Dementia Unit at Consorci Sanitari de Terrassa.

To calculate the sample size we used G*Power software (v 3.1.0.2) [35, 36], assuming a dropout risk of a 20% (so the withdrawal of participants does not undermine the clinical relevance of the results), a type I error probability (α) of 0.05, and a type II error probability or statistical power (1 − β) of 0.8. The effect size for a treatment consisting of CS is medium [37] and, given the recent results [5, 19] we expect TMS to increase the effects of CS alone, then assuming a Cohen’s effect size of at least 0.6. Thus, the total recommended sample size is 45, 15 per arm to which we added 3 more participants per group to cover the 20% dropout risk. The risk of clustering effect [38] is absent in this trial since the centre and the healthcare professionals providing the treatments (TMS and/or CS) will be the same for all patients.

### Procedure

The intervention consists of a 2-week treatment during which TMS will be applied for 10 days over four different brain regions (see below). Since both, short single-region interventions and long multiple-region interventions have achieved positive outcomes in terms of cognitive and functional improvements [e.g., 13, 15, 18, 39] we have followed a cost-effectiveness approach proposing a short (2 weeks) multi-region (four brain areas) intervention to maximize the outcomes while reducing the costs. The stimulation protocol will be the iTBS (600 pulses in bursts of 3 pulses applied at 50 Hz administered every 200 ms -5 Hz- with intervals of 2 s of stimulation and 8 s of rest, lasting 3 min and 12 s). The stimulation will be delivered using a Magstim Super Rapid2 device, with a 70 mm, 8-figure coil and neuronavigated using Brainsight™ 2 device. The stimulation intensity will be set at the 80% of the active motor threshold [see safety guidelines, 10, 34].

Stimulation will be delivered over the DLPFC and the PC in both hemispheres (1 day left DLPFC and right PC with a 15 min interval, and the contralateral areas the following day). The specific target areas for stimulation will be determined individually based on the functional connectivity of each area with two subcortical regions related to AD cognitive dysfunction: the fornix and the hippocampus respectively [40–43]. The seeds used to compute the connectivity analysis, will be 10 mm radius spheres placed bilaterally on the fornix and the hippocampus and adjusted to individual anatomical landmarks. The selection of the specific TMS targets within the DLPFC and PC will be based on its functional connectivity with the seeds. Based on previous literature, the stimulation over the DLPFC is intended to improve participants performance in language tasks and general functioning [11, 13, 14] while stimulation in parietal is intended to improve performance in memory tasks [43, 44].

The placebo condition (sham TMS control group) will be performed using the same stimulation protocol as the active condition over the same areas (bilateral DLPFC and bilateral PC) but with the coil rotated 90° to prevent the magnetic field from inducing electrical activity in the cortex.

Before and after each session, mood and fatigue will be assessed using a visual analogue scale (VAS). At the end of each session, the side effects of TMS will be also assessed.

Along with the TMS treatment, all patients will undergo the CS intervention programme regularly provided by the Consorci Sanitari de Terrassa. The CS is based on Clare and Woods’ definition [45] and Bottino’s et al. model [46], and follows the basic principles of non-pharmacological interventions aiming to improve the quality of life through engagement in significant activities. The program includes 1-h cognitive stimulation and occupational therapy group sessions three times per week (10–12 patients per group). All sessions are conducted by an occupational therapist and supervised by a clinical neuropsychologist who design the CS for each patient. All sessions include reality orientation therapy for 10 min, and training in attention and concentration, memory, language, calculation, gnosias, praxias, or executive function for 50 min [for more details see, 47].

**Fig. 1:**
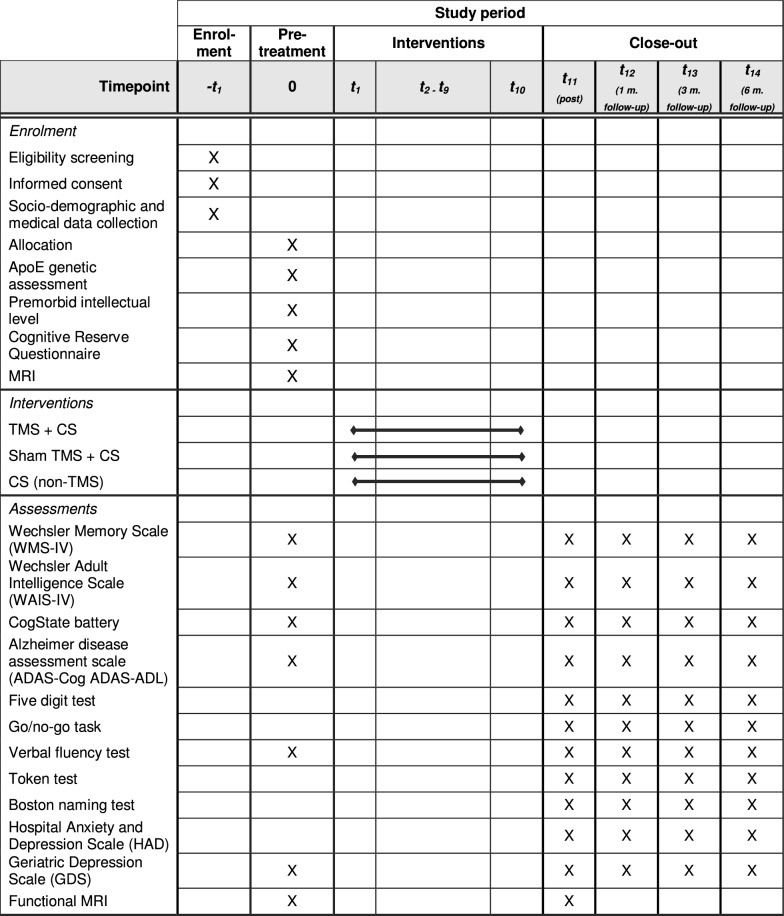
Trial timeline following SPIRIT recommendations

Neuropsychological, functional and emotional aspects will be assessed before and after the intervention (see Table 2 and Fig. 1). A neuropsychologist blinded to the treatment will manage the outcome measurements at all intervention time points.

**Table 2:** Outcome measures

*Primary outcome measures*	
Memory	• Logical Memory, Wechsler Memory Scale IV (WMS-IV) [48] • International Shopping List Task • One Card Learning Task of the neurocognitive computerized battery CogState [49, 50]
Attention	• Identification Task of CogState computerized battery [49, 50] • Direct digits of the Wechsler Adult Intelligence Scale IV (WAIS-IV) [51]
Working memory	• Ad hoc computerized Zero and One-Back task and the subtest of the backward digit span test of WAIS-IV [51]
Executive functions	• Five Digit Test [52] • Ad hoc computerized Go/No-Go task • Verbal fluency test (letters P-M-R and animals) [53]
Language	• Token Test [54] • Short form of the Boston Naming Test [55]
Processing speed	• Detection Task of CogState computerized battery [49, 50]
General cognitive	• Alzheimer’s Disease Assessment Scale – cognitive subscale (ADAS-Cog) [56]
*Secondary outcome measures*	
Functional connectivity	• Assessed one time after 10 treatment sessions, through the registry of brain activity in resting state MRI
Functional capacity	• Functional Assessment Questionnaire (FAQ) [57] • UCSD Performance-Based Skills Assessment (UPSA) [58]
Mood changes (depression)	• Hospital Anxiety and Depression scale (HAD) [59] • Geriatric Depression Scale (GDS) [33]
Activities of daily living	• Alzheimer’s Disease Assessment Scale – activities of daily living subscale (ADAS-ADL) [56]
*Modulatory outcome measures*	
Premorbid intellectual level	• “Word Accentuation Test” (a Spanish language test) [60]
Cognitive reserve	• Cognitive Reserve Questionnaire [61]
ApoE (ε4, ε2)	• Genetic analysis

Given the need of multiple administrations, we avoided neuropsychological tests with a marked practice effect, and selected, when possible, tests with parallel versions. The length of each assessment session is ~ 2 h.

All participants will undergo an MRI scan before and after their participation in order to: (1) detect the presence of neurological disorders; (2) localize individual cortical targets for TMS based on their functional involvement in cortico-subcortical networks; (3) guide neuronavigated TMS; and (4) assess the functional and structural brain changes after the intervention.

First exploration, lasting ~ 30 min, will consist in a brain volumetric acquisition (3D) and a resting state acquisition. At the end of the study (three days after treatment), resting state will be acquired again to obtain reliable data on the effects of the intervention on brain activity.

The planning of the clinical trial following the SPIRIT guidelines is displayed in Fig. 1.

### Ethical and legal aspects

Patients’ participation will be voluntary after being informed about the objectives of the study and signing an informed consent form. The participants will be free to withdraw from the study at any time. The researchers agree to respect all the established current legislation regarding clinical research (WMA Declaration of Helsinki, 2004; Law 41/2002 on patient autonomy). The Institutional Review Board of the Consorci Sanitari de Terrassa has approved this project.

In accordance with Regulation (EU) 2016/679, on the protection of personal data, any data collected from the participants will be treated with strict confidentiality.

### Possible risks, side effects and discomforts

TMS has been used in research for more than 20 years and safety guidelines have been developed [62]. In this study, all the safety recommendations will be followed and a doctor will always be on call during the TMS sessions.

### Statistical analyses

Statistical analyses will be performed using SPSS (v. 23). We will perform a descriptive analysis of demographic and clinical variables (age, sex, years of schooling, diagnosis, and comorbid disorder) and multivariate analysis of variance for repeated measures of cognitive, emotional and functional variables included as a measure of efficacy. All statistical tests will be performed using a significance level of 0.05.

For processing and analysis of magnetic resonance images we will use different software packages: FSL (FMRIB Software Library, www.fmrib.ox.ac.uk/fsl), and FreeSurfer (http://surfer.nmr.mgh.harvard.edu).

### Discussion

Nowadays, AD is the most common cause of dementia with no known cure. The cognitive decline increase as the disease progresses, and existing therapeutic approaches are not efficient in the improvement of cognitive deficits or functional limitations. TMS seems to be a promising tool for this purpose, given its ability to modulate cortical excitability and neural network activity.

Although research in this field has notably increased in recent years, it is still very scarce and the most effective stimulation parameters in terms of frequency, intensity, localization and length of stimulation, are unknown. Additionally, it is necessary to include functional and structural neuroimaging measurements to reveal the underlying neural mechanisms of the beneficial effects of TMS.

The expected results of this research will contribute to deepening the knowledge of the effectiveness of TMS as a therapeutic approach in AD, one of the most prevalent, disabling and incapacitating diseases nowadays.

## Limitations

The main limitation of this study is the heterogeneity of AD patients. The variability in clinical symptoms can hinder the capacity to extract robust findings from clinical trials. To avoid this risk, a wide range of inclusion and exclusion criteria have been established. This strategy also comprises another limitation related to the recruitment process to achieve the required sample size. Thus, the strict exclusion criteria will prolong the recruitment process but it will ensure the detection of clinically meaningful effects.

Finally, another major possible limitation will be the experimental mortality due to the length of the study, which includes two follow-up assessments 1, 3, and 6 months after the intervention.

### Trial status

This trial has not started the patient recruitment phase yet since no funding has been obtained to date.

## Abbreviations

AD: Alzheimer’s disease
CS: cognitive stimulation
DLPFC: dorsolateral prefrontal cortex
iTBS: intermittent theta burst stimulation
MRI: magnetic resonance imaging
PC: parietal cortex
TMS: transcranial magnetic stimulation
